# The Birth and Demise of the IS*Apl1*-*mcr-1*-IS*Apl1* Composite Transposon: the Vehicle for Transferable Colistin Resistance

**DOI:** 10.1128/mBio.02381-17

**Published:** 2018-02-13

**Authors:** Erik Snesrud, Patrick McGann, Michael Chandler

**Affiliations:** a Multidrug-Resistant Organism Repository and Surveillance Network, Walter Reed Army Institute of Research, Silver Spring, Maryland, USA; b Laboratoire de Microbiologie et Genetique Moleculaires, Centre National de la Recherche Scientifique, Toulouse, France; c Department of Biochemistry, Molecular and Cellular Biology, Georgetown University Medical Center, Washington, DC, USA; Skirball Institute of Biomolecular Medicine, New York University Medical Center

**Keywords:** colistin resistance, composite transposon formation, drug resistance evolution, insertion sequence, transposon decay

## Abstract

The origin and mobilization of the ~2,609-bp DNA segment containing the mobile colistin resistance gene *mcr-1* continue to be sources of uncertainty, but recent evidence suggests that the gene originated in *Moraxella* species. Moreover *mcr-1* can be mobilized as an IS*Apl1*-flanked composite transposon (Tn*6330*), but many sequences have been identified without IS*Apl1* or with just a single copy (single ended). To further clarify the origins and mobilization of *mcr-1*, we employed the Geneious R8 software suite to comprehensively analyze the genetic environment of every complete *mcr-1* structure deposited in GenBank as of this writing (September 2017) both with and without associated IS*Apl1* (*n* = 273). This revealed that the 2,609-bp *mcr-1* structure was likely mobilized from a close relative of a novel species of *Moraxella* containing a chromosomal region sharing >96% nucleotide identity with the canonical sequence. This chromosomal region is bounded by AT and CG dinucleotides, which have been described on the inside ends (IE) of all intact Tn*6330* described to date and represent the ancestral 2-bp target site duplications (TSDs) generated by IS*Apl1* transposition. We further demonstrate that all *mcr-1* structures with just one IS*Apl1* copy or with no IS*Apl1* copies were formed by deletion of IS*Apl1* from the ancestral Tn*6330*, likely by a process related to the “copy-out–paste-in” transposition mechanism. Finally, we show that only the rare examples of single-ended structures that have retained a portion of the excised downstream IS*Apl1* including the entire inverted right repeat might be capable of mobilization.

## IMPORTANCE

A comprehensive analysis of all intact *mcr-1* sequences in GenBank was used to identify a region on the chromosome of a novel *Moraxella* species with remarkable homology to the canonical *mcr-1* structure and that likely represents the origin of this important gene. These data also demonstrate that all *mcr-1* structures lacking one or both flanking IS*Apl1* were formed from ancestral composite transposons that subsequently lost the insertion sequences by a process of abortive transposition. This observation conclusively shows that mobilization of *mcr-1* occurs as part of a composite transposon and that structures lacking the downstream IS*Apl1* are not capable of mobilization.

## INTRODUCTION

Over the past decades, the serious threat to public health posed by the development and rapid spread of antibiotic resistance (Ab^r^) has become increasingly clear to the medical community and to the general public (1). This dissemination of resistance genes is facilitated by their frequent sequestration into transposable genetic elements (TE), small DNA segments capable of moving from one place in their host genome to another. Insertion of TE into transmissible plasmids subsequently facilitates their colonization of other bacteria of the same species and, depending on the plasmid, of other species and genera.

There are several ways in which Ab^r^ genes may be sequestered into TEs (see reference 2). Among the important Ab^r^ gene sources are composite or compound transposons frequently found in nature (3). Establishment of compound structures is thought to result from random insertion of two IS copies on either side of an Ab^r^ passenger gene. However, there are examples of structures which include an entire flanking IS copy at one side and a surrogate IS end located at some distance at the other (4–7). The way in which such structures arise and subsequently decay has not been documented. However, the massive increase in DNA sequencing power has now provided us with the tools necessary to investigate transposon populations and the way in which transposons and plasmids change over time within and between bacterial populations (and their hosts). This allows us to address issues of short-term evolution of these highly plastic mobile genetic elements: how they arise and how they decay over time.

A recently identified example of the birth and decay of a compound transposon concerns the *mcr-1* gene responsible for resistance to the last-resort antibiotic, colistin (polymyxin E). Identification of this transferable phosphoethanolamine (pEtN) transferase gene in November 2015 caused great concern (8). Prior to the discovery of *mcr-1*, colistin resistance mediated through pEtN modification of lipid A was attributed primarily to mutations in regulators of intrinsic pEtN transferases such as the PmrAB system in *Acinetobacter baumannii* (9) or the PhoPQ system in *Klebsiella pneumoniae* (10). Although they perform the same function, there is considerable diversity among pEtN transferases (8).

The origin of *mcr-1* is still uncertain. In most *mcr-1* copies identified to date, the region encompassing this gene is an ~2,609-bp DNA segment containing *mcr-1* and a putative 765-bp open reading frame (ORF) gene encoding a protein similar to a PAP2 superfamily protein (8). The initial description of MCR-1 indicated that it aligned to pEtN from several species, including *Moraxella catarrhalis*, with which it shared 59% amino acid identity (8). More recently, AbuOun and colleagues reported a chromosomal region on *Moraxella* sp. MSG13-C03 that shared 96.6% nucleotide identity (NI) with the 2,609-bp *mcr-1*-*pap2-*containing DNA segment (11). However, irrespective of the origins of this gene, the initial mobilization of *mcr-1* appears to be closely associated with insertion sequence (IS) IS*Apl1* (12), a member of the IS*30* insertion sequence family, most likely via the composite transposon Tn*6330* (13).

**FIG 1:**
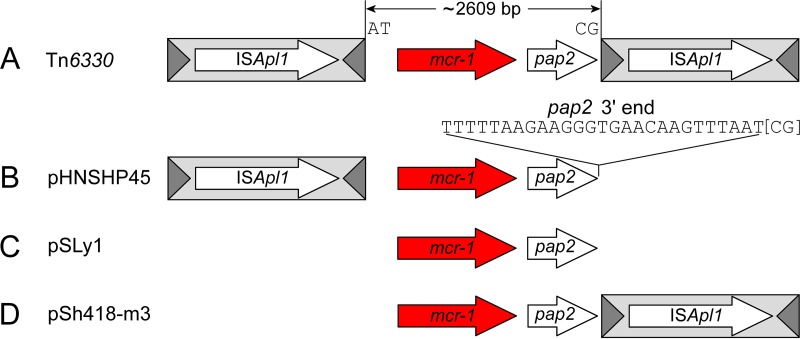
Four representative sequences showing the four general *mcr-1* structures identified to date. (A) The composite transposon Tn*6330* (13). (B) pHNSHP45, a single-ended structure with an upstream copy of IS*Apl1* only. (C) pSLy1, a structure lacking both copies of IS*Apl1*. (D) pSh418-m3, a single-ended structure with a downstream copy of IS*Apl1* only. The ~2,609-bp region comprising the *mcr-1* region is shown flanked by the conserved, ancestral TSD dinucleotides AT and CG on the IE. The last 27 bp of the *mcr-1* region that comprise the 3′ end of the putative *pap2* gene are indicated for clarity. IS*Apl1*, light gray box; transposase, long white arrow; terminal inverted repeats, dark gray triangle; *mcr-1*, red arrow; *pap2*, short white arrow. The same scheme is used for all of the figures.

We showed previously (12) that *mcr-1* sequences deposited in public databases have three general structures (Fig. 1): that of IS*Apl1* composite transposon Tn*6330* with directly repeated flanking IS copies (13) (Fig. 1A); those with just a single, upstream IS*Apl1* copy (single ended) (Fig. 1B); and sequences lacking IS*Apl1* altogether (Fig. 1C). However, in some of the latter two types of structure, the remnants of an ancestral IS*Apl1* were still present (12). Recently, Poirel et al. demonstrated mobilization of the *mcr-1* fragment by an engineered derivative of Tn*6330*, Tn*6330*.*2*, experimentally confirming that Tn*6330* is a primary vehicle for *mcr-1* transposition (14).

Much of the uncertainty surrounding the role of IS*Apl1*-mediated *mcr-1* mobilization has centered on the single-ended variants, with some studies suggesting that *mcr-1* can be mobilized by this structure (15, 16). Gao and colleagues postulated that this is achieved as a consequence of the presence of a pseudo-IS*Apl1* inverted right repeat (IRR) that encompasses the last 26 bp of the *pap2* gene (Fig. 1) (15). Putative target site duplications (TSDs) were identified in a number of single-ended variants, but it is unclear whether these are true TSDs. Since IS*Apl1* generates only a short, 2-bp TSD upon integration, determining whether these represent true TSDs or are simply serendipitous is difficult (12). Indeed, recent experiments failed to detect *mcr-1* transposition by this type of single-ended variant with the pseudo-IS*Apl1* IRR (14), although the frequencies obtained for transposition of Tn*6330*.*2* itself were extremely low and the frequency of transposition of a transposon with a pseudoend might be expected to be even lower.

In the present study, we conducted a comprehensive analysis of all *mcr-1*-containing sequences deposited in GenBank to date (*n* = 273; see Table S1 in the supplemental material). Our data suggest that Tn*6330* was generated by IS*Apl1* capture of a *mcr-1* gene from a close relative of *Moraxella* sp. MSG13-C03 whose genomic sequence was recently deposited in GenBank (11). Furthermore, our data indicate that single-ended variants of Tn*6330* are the result of IS*Apl1* loss and appear capable of transposition only when a fully intact IRR of this ancestral IS*Apl1* is present. Finally, the results show that IS*Apl1* is exceptionally recombinogenic in natural populations. Tn*6330* has a propensity to lose one or both flanking IS*Apl1* copies, resulting in loss of transposability and stabilization of the *mcr-1* gene. In addition to the three structures described previously (12), we have now observed a rare fourth structure in which the upstream copy of IS*Apl1* is deleted while the downstream copy is retained (Fig. 1D). This type of deletion activity had also been observed for the founding member of this IS family, IS*30*, in the laboratory (17). Finally, to explain the frequent IS*Apl1* deletions, we provide a model that is based on the transposition mechanism of IS*30* family members (18) and which invokes a process of abortive transposition.

10.1128/mBio.02381-17.4 TABLE S1  Information concerning the sequences analyzed. Column 1, GenBank accession numbers of the sequences; column 2, location of the sequence (in either plasmid or chromosome); column 3, plasmid name (where applicable); column 4, plasmid incompatibility group (Inc) (where known); column 5, sequencing platform; column 6, source of the sequence, organism, and strain; column 7, type of sequence (composite transposon with two flanking IS; double deletion in which both flanking IS have been excised; single deletion in which the left or right IS copy has been excised; empty site where an identical sequence without the transposon insertion was identified); column 8, the 2-bp target site duplication (TSD); column 9, unique insertion site code; column 10, specific comments (where applicable). Download TABLE S1, XLSX file, 0.03 MB. Copyright © 2018 Snesrud et al. 2018 Snesrud et al. This content is distributed under the terms of the Creative Commons Attribution 4.0 International license.

## RESULTS

To gain greater insight into how IS*Apl1* has mobilized *mcr-1*, we undertook an extensive *in silico* analysis of all interpretable *mcr-1* sequences deposited in GenBank as of this writing (*n* = 273; September 2017) (see Table S1 in the supplemental material). All sequences share an ~2,609-bp DNA segment (Fig. 1A) (8) containing *mcr-1* and a putative 765-bp *orf*, *pap2*, encoding a Pap2 superfamily protein. There is some minor variability in the length of this *mcr-1* region due to the presence of small insertions/deletions and mutations at the extremities, but, apart from this, the sequences are highly conserved. Variability in the immediate flanking regions is primarily due to the presence or absence of IS*Apl1*. In the analysis presented below, we address how these structures may have been formed and their relationship to each other.

### The birth of Tn*6330*: IS*Apl1-mcr-1-*IS*Apl1*.

**FIG 2:**
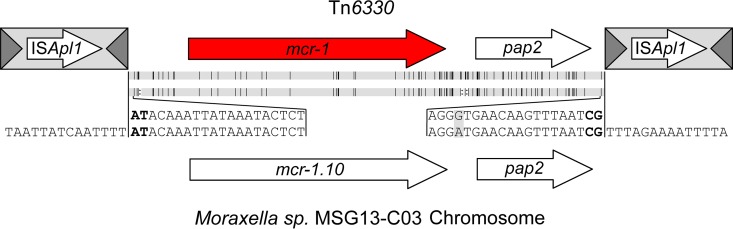
Alignment of Tn*6330* and the homologous region on the *Moraxella* sp. MSG13-CO3 chromosome. The consensus sequence at the end of both regions is provided, with the conserved AT and CG dinucleotides that are found on the IE of all Tn*6330* highlighted in bold. Mutations and deletions are indicated by vertical black lines and colons, respectively, between the two images.

During our analysis of *mcr-1* sequences for this study, we identified a recent GenBank submission of a nucleotide sequence termed *mcr1.10* (MF176238) that showed 97.6% identity to the canonical *mcr-1* gene (11). The gene was found in *Moraxella* sp. MSG13-C03 and was present in a 2,606-bp chromosomal region that shared a strikingly high level of nucleotide identity (96.6%) with the 2,609-bp *mcr-1* region (Fig. 2). Furthermore, as shown by calculation of the average score of all substitutions in the alignment using a BLSM62 substitution-scoring matrix (19), *mcr-1* aligned to this region with a pairwise positive-identity level of 99.3%. *Moraxella* sp. MSG13-C03 was isolated in April 2014 in the United Kingdom from the cecal contents of a healthy pig (11). Analysis indicates that *Moraxella* sp. MSG13-C03 represents a novel species whose closest relatives are *Moraxella porci* DCM 25326 (GenBank accession number NZ_MUYV00000000.1) and *Moraxella pluranimalium* CCUG 54913 (GenBank accession number NZ_MUYU00000000.1), with which it shares average nucleotide identities (ANI) of 82.3% and 81.3%, respectively (Fig. S1). The 2,606-bp *Moraxella* sp. MSG13-C03 region encompassing the *mcr-1* and *pap2* genes has 3 indels compared to the canonical 2,609-bp *mcr-1* structure, but the missing bases are all in noncoding regions: One occurs upstream of *mcr-1*, while the others occur between *mcr-1* and the start of *pap2* (Fig. 2). Of particular note, this 2,606-bp region is flanked upstream by the dinucleotide AT and downstream by the dinucleotide CG (11) (Fig. 2, bold text). The same nucleotides were previously reported to occur at the inside ends (IE) of the flanking IS*Apl1* copies in Tn*6330*, likely representing the ancestral TSDs formed during the initial mobilization of this gene by IS*Apl1* insertion (12).

10.1128/mBio.02381-17.1 FIG S1  Phylogenetic tree generated from the core genome sequences of various *Moraxella* species using RAxML (34). The genome of *Moraxella* sp. strain MSG13-C03 is highlighted in blue. Branch lengths are proportional to the number of nucleotide changes with 100 bootstrap replications. Download FIG S1, TIF file, 2.5 MB. Copyright © 2018 Snesrud et al. 2018 Snesrud et al. This content is distributed under the terms of the Creative Commons Attribution 4.0 International license.

The presence of these dinucleotides bounding a region of such high homology to the *mcr-1* region strongly suggests that the initial mobilization of *mcr-1* occurred from a *Moraxella* species closely related to *Moraxella* sp. MSG13-C03 by insertion of IS*Apl1* upstream and downstream. IS*Apl1* insertion into these T-A-rich target sites flanking the 2,606-bp region (Fig. 2) presumably generated the 2-bp TSDs AT and CG at the directly repeated upstream and downstream IS*Apl1* copies, respectively. This would have constituted the ancestral Tn*6330*. In further transposition events, the ancestral TSDs located at the Tn*6330* outside ends (OE) would be replaced by new TSDs generated by Tn*6330* insertion into a new target site. The resulting IS*Apl1* composite transposon, Tn*6330*, would retain both internal conserved 2-bp fingerprints (AT and CG).

### IS*Apl1-mcr-1-*IS*Apl1* composite transposons (Tn*6330*) have target site duplications.

As of this writing (September 2017), 31 *mcr-1* sequences with complete directly repeated flanking IS*Apl1* copies forming the composite transposon Tn*6330* had been deposited in GenBank (Table S1). Twenty-two were located on plasmids encompassing a variety of incompatibility (Inc) classes and 9 on host chromosomes (Table S1). There is some redundancy in this collection, as only 20 different insertion sites were identified from the 31 sequences. In each case, the conserved ancestral AT and CG dinucleotides are evident at the IE of the flanking IS*Apl1* (Fig. 1A). In the majority of cases, Tn*6330* is flanked by 2-bp TSDs, characteristic of IS*Apl1* transposition. Six Tn*6330* derivatives lack these TSDs, but all are associated with plasmid rearrangements that have resulted in TSD deletion after Tn*6330* insertion (Fig. S3). Furthermore, the sequences of Tn*6330* were >99% identical in all of the structures and therefore appear to be active representative copies of a single ancestral transposon.

Additional genetic events within Tn*6330* were also evident among seven sequences from five unique insertions (Table S1). Tn*3* and IS*Kpn26* have inserted into Tn*6330* in pMCR_1511 and pls1, respectively, generating characteristic 5-bp (Tn*3*; GTAAA) and 4-bp (IS*Kpn6*; CTAG) TSDs (data not shown). Similarly, IS*1294* has inserted into Tn*6330* in five sequences representing 3 different insertion sites (Table S1). No TSDs are evident with these insertions because IS*1294* transposes via a rolling-circle mechanism and does not generate TSDs upon integration (20).

### Tn*6330* derivatives which retain a functional downstream IS*Apl1* IRR.

**FIG 3:**
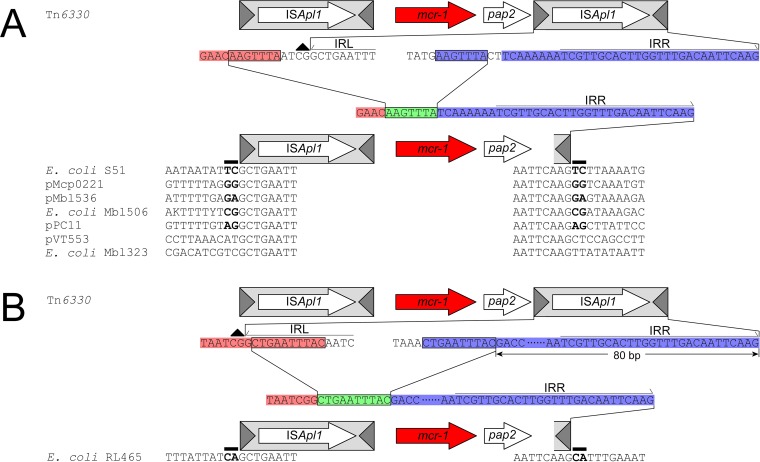
Schematic representation of single-ended *mcr-1* cassettes that have retained the last 42 bp (A) or 90 bp (B) of an ancestral IS*Apl1* that includes the entire IRR. The conserved, ancestral CG dinucleotide on the IE of the downstream IS*Apl1* is indicated with a black triangle. The bases upstream and downstream of the deletion that are retained after IS*Apl1* loss are highlighted in red and blue, respectively. The deletion joints upstream and downstream of the IS*Apl1* are encased in a black rectangle. The bases upstream and downstream of the deletion that are retained after IS*Apl1* loss are highlighted in red and blue, respectively, while the remaining copy of the deletion joint that is retained after the two ends are joined following IS*Apl1* excision is highlighted in green and encased in a black rectangle. The same labeling scheme is used throughout the figures. Putative 2-bp TSDs in 5 of the 7 sequences are highlighted in bold.

There are 10 single-ended derivatives in the library (Table S1) with a deletion within the downstream IS*Apl1* but where a significant segment of the right IS*Apl1* end is retained, including an entire 27-bp IRR presumably essential for transposase recognition and activity. One of these had been identified previously (12) in the *Escherichia coli* RL465 chromosome (LT594504). This retained the last 90 bp of the IS. This structure is flanked by a putative 2-bp TSD (CA). A second, identified in the *E. coli* S51 chromosome, retained 42 bp of the IS*Apl1* right end, including the entire IRR (Table S1) (12). This structure is also flanked by a probable 2-bp TSD (TC). We had previously speculated that these may be mobile due to the presence of the intact IRR ends and the putative TSDs (12). Since then, 7 additional sequences, representing 5 unique insertion sites, which also retain the last 42 bp of an ancestral downstream IS*Apl1* have been deposited in GenBank (Table S1) (Fig. 3). Three are present on host chromosomes, and 4 are located on IncI2 or IncK2 plasmids (21, 22). Four have putative TSDs flanking the entire structure, and at least three of these appear to be legitimate based on a comparison with corresponding empty site sequences (i.e., identical sequences with no *mcr-1* insertion). In the remaining 3 structures, no TSDs are present, but deletions and rearrangements similar to those observed with some of the composite transposon structures are evident in the surrounding area which likely removed the TSDs. On the basis of these additional data, it is possible that single-ended variants retaining the IRR of an ancestral downstream IS*Apl1* are capable of transposition, though experimental verification would be required to address this definitively.

### IS*Apl1*-*mcr-1*: single-ended Tn*6330* variants were created by the loss of a downstream IS*Apl1.*

The members of a second major class of 59 IS*Apl1*-associated *mcr-1* genes have only a single, upstream IS*Apl1* copy (Fig. 1B). Fifty sequences are located on plasmids representing a variety of Inc types, 8 on host chromosomes, and 1 in a putative phage. There is significant redundancy within these 59 sequences, however, and just 18 unique insertion sites are present (Table S1). In all 59 sequences, the ~2,609-bp regions encompassing *mcr-1* and *pap2* are >99% identical except for a concentration of nucleotide variability at the 3′ end of *pap2*.

Putative 2-bp TSDs could be identified flanking 29 sequences representing 10 unique insertions sites, and this has led to speculation that these structures are mobile, ostensibly due to an imperfect IRR encompassing the last 26 bp of *pap2* (Fig. 1B) (15). There are two plausible hypotheses to explain these: (i) they are mobile and have transposed using the imperfect IRR (15) or (ii) they represent Tn*6330* decay products in which the downstream IS*Apl1* copy has undergone deletion.

**FIG 4:**
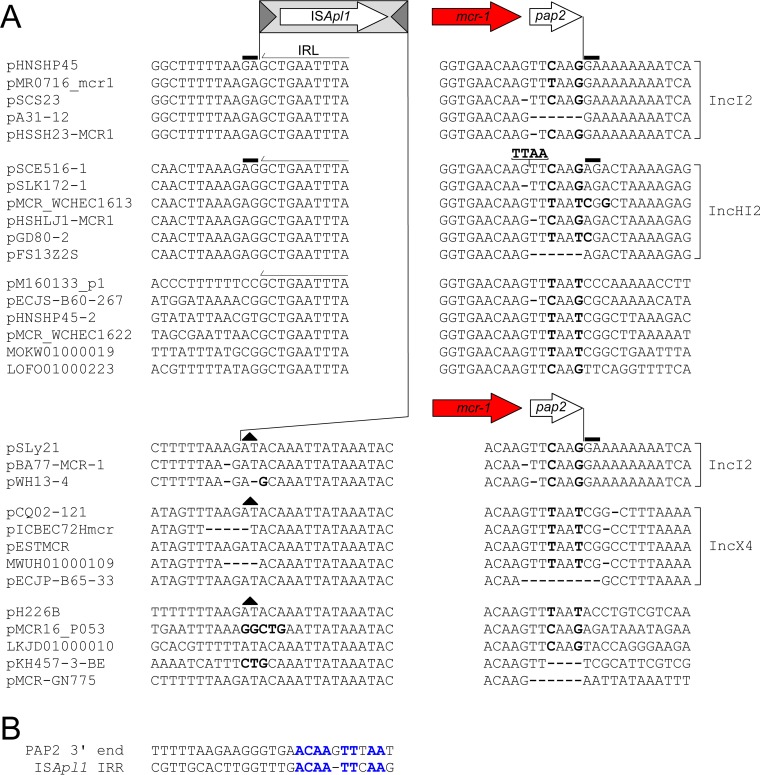
(A) Consensus alignment of multiple *mcr-1* regions from a variety of plasmid backbones with one (single-ended) flanking copy or no flanking copy of IS*Apl1*. The minor sequence variation at the 3′ end of the *pap2* gene in all structures and at the 5′ end of *mcr-1* in structures lacking the upstream IS*Apl1* is highlighted in bold text, with absent bases indicated by bold dashes. To preserve the alignment, the additional TTAA tetranucleotide in pSCE516-1 has been placed above the sequence, with a line indicating the correct position. (B) Alignment of the last 27 bp of the *pap2* gene and the 26 bp that constitute the IRR of IS*Apl1*. The nucleotides that form the basis for the sequence variability noted in structures that have lost the downstream IS*Apl1* are highlighted in blue.

A notable feature of all single-ended variants, irrespective of whether putative TSDs are present, is a concentration of nucleotide polymorphisms at the 3′ end of the *pap2* gene (12) (Fig. 4; bold text). For example, an alignment of the single-ended variants in many IncI2 plasmids suggests that a GA TSD flanks the entire structure (Fig. 4; black rectangle). However, when the sequences directly upstream of this putative TSD were aligned, some contained a CAAG tetranucleotide, others had a TAAG, and others appeared to be missing one or more bases (Fig. 4; bold text). This pattern is repeated throughout other single-ended variants even in plasmids that are almost identical. In analyzing these base changes, we noted a pattern in the nucleotide differences that was consistent with the second hypothesis proposed above: deletion of an ancestral downstream IS*Apl1*. In the majority of single-ended variants, an ancestral downstream IS*Apl1* was found to have been removed from a position between an ~20-bp upstream region that encompasses the 3′ end of *pap2* and an ~20-bp downstream region encompassing the 3′ end (i.e., the IRR) of an ancestral downstream IS*Apl1* (Fig. 4B; bold blue). This fully explains all nucleotide variation in single-ended variants. Excision of IS*Apl1* between these regions occurs at deletion joints that range in length from 1 to 4 bp and which we hypothesize to be the point at which the two ends are joined after IS*Apl1* excision.

To better clarify this, we identified complete Tn*6330* copies on plasmid backbones that were highly similar to those carrying the single-IS*Apl1* sequences and that were also inserted into the same location. Furthermore, we also identified highly similar plasmids with corresponding empty sites allowing us to accurately examine the nucleotide variation present at the 3′ end of the single-ended variants.

**FIG 5:**
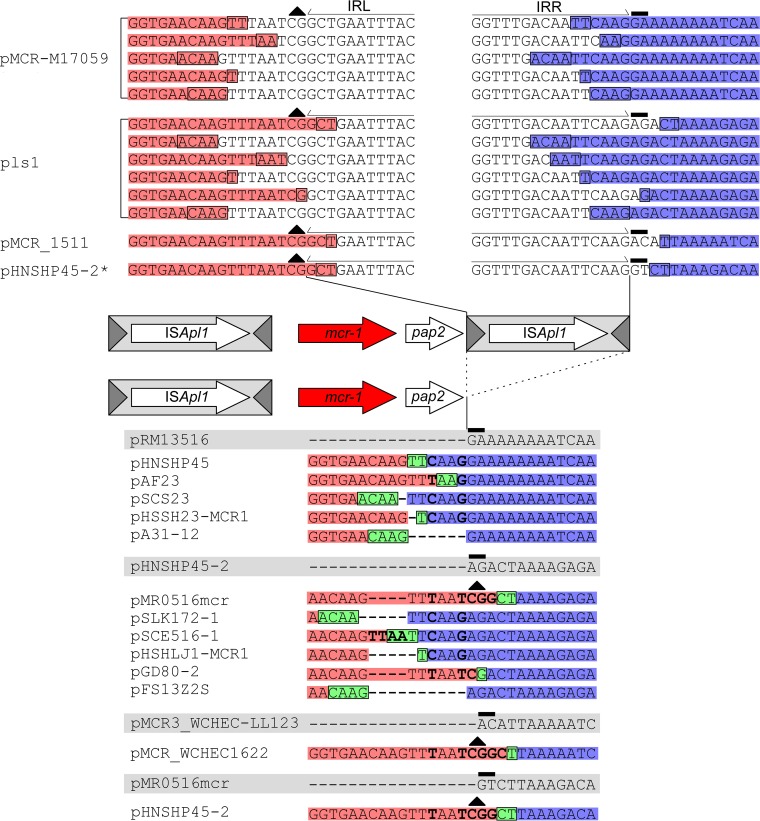
Alignment showing the decay of multiple different instances of Tn*6330* (top) into the corresponding single-ended structures formed (bottom of figure), aligned with their respective empty sites (bottom of figure, highlighted in gray; see the text for more details). “Hypothetical” Tn*6330* insertions constructed from known empty site plasmids, the corresponding single-ended structures, and the sequence of Tn*6330* are indicated with an asterisk. The labeling scheme is identical to that described for Fig. 3.

The top of Fig. 5 shows multiple examples of a variety of insertion sites and plasmids with representative composite transposons deposited in GenBank. In addition, we inferred the sequence of a hypothetical parental composite transposon insertion for pHNSHP45-2 (marked with an asterisk), which has had no corresponding composite transposon deposited to date. This was accurately achieved by comparing the single-ended sequence, its corresponding empty site, and known sequences of other composite transposons. The 2-bp TSDs flanking the downstream IRR that was generated upon insertion of the original composite transposon (prior to the loss of the downstream copy) are indicated (black, solid rectangle above the dinucleotide). The conserved, ancestral CG dinucleotide abutting the left inverted repeat (IRL) on the IE of the downstream IS*Apl1* is indicated (black triangle). The bottom of Fig. 5 illustrates the single-ended variants formed through loss of the downstream IS*Apl1* identified in our library. These are compared to the real (and, for pHNSHP45-2, “hypothetical”) composite transposons described above, with the deletion junction encased in a black rectangle and highlighted in green. Nucleotides highlighted in red represent the original sequences upstream of the excision point that are retained in the single-ended variant, while those highlighted in blue represent the sequences retained downstream of the excision. The corresponding empty site for each sequence is highlighted in gray, with the 2-bp TSD that was generated upon insertion of the ancestral Tn*6330* highlighted by a black, solid rectangle above the dinucleotides. The nucleotides that form the basis for the variation noted above at the 3′ end of the single-ended structures are highlighted in bold and can be consistently explained by small variations in the region excised during loss of IS*Apl1*. It is noteworthy that, although there are some minor variations in the excision point, the length of the excised region ranges from 1,064 bp to 1,074 bp, with an average of 1,070 bp (standard deviation [st. dev.], 2.2 bp; Fig. S2), the same length as IS*Apl1*. For example, if the excision occurs further into the *pap2* gene (i.e., further in the 3′ direction), the point of the downstream excision has a tendency to shift in the 3′ direction to compensate, and vice versa.

**FIG 6:**
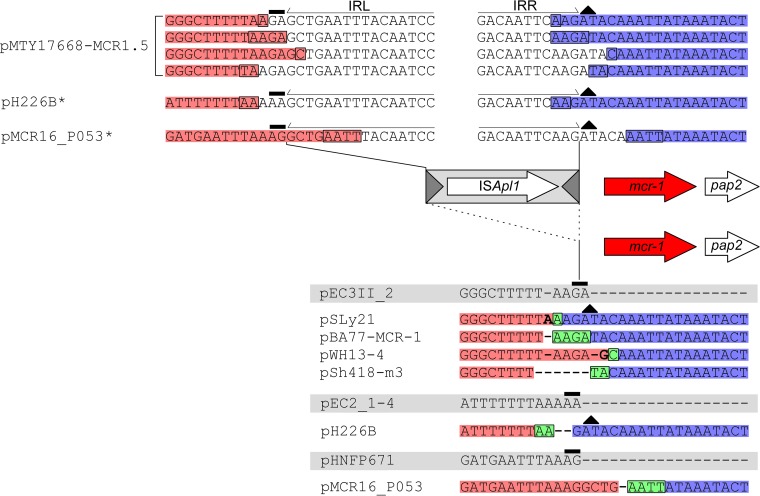
Alignment showing the decay of multiple different instances of Tn*6330* (top) into the corresponding double-deletion structures formed (bottom of figure), aligned with their respective empty sites (below, highlighted in gray; see the text for details). “Hypothetical” Tn*6330* insertions constructed from known empty site plasmids, the corresponding double-deletion structures, and the sequence of Tn*6330* are indicated with an asterisk. Dashes were inserted to maintain sequence alignment.

10.1128/mBio.02381-17.2 FIG S2  Distribution of the different deletion sizes following loss of the downstream IS*Apl1* in all 19 sequences shown in Fig. 5 and 6. The average deletion size is 1,069.8 bp (standard deviation, 2.4). Download FIG S2, TIF file, 7.9 MB. Copyright © 2018 Snesrud et al. 2018 Snesrud et al. This content is distributed under the terms of the Creative Commons Attribution 4.0 International license.

10.1128/mBio.02381-17.3 FIG S3  Comparison of plasmids with Tn*6330* insertions to the corresponding “empty site” plasmids, indicating the various events that have removed the TSD in some Tn*6330* structures. (A) The deletion of ~6,380 bp from the pECJP-59-244 plasmid backbone compared to empty site plasmid p180-PT54. (B) The deletion of ~1,480 bp from the P14408_M1 and pSA-MCR-1 plasmid backbones with a concomitant inversion of the downstream IS*Apl1* gene compared to empty site plasmid p180-PT54. (C) The deletion of ~1,450 bp from the plasmid backbone in pS38 with a corresponding movement of the last 36 bp of the downstream IS*Apl1* directly upstream of the IRL of the upstream IS*Apl1*. The *mcr-1* and *pap2* genes are represented by a red block arrow and a white block arrow, respectively. Truncated genes are represented by ragged ends. The solid, rotated triangles at the ends of IS*Apl1* represent the IRL (left) and IRR (right) sequences. Download FIG S3, TIF file, 5.5 MB. Copyright © 2018 Snesrud et al. 2018 Snesrud et al. This content is distributed under the terms of the Creative Commons Attribution 4.0 International license.

### IS*Apl1* loss resulted in *mcr-1* regions entirely devoid of IS*Apl1.*

By far the largest numbers of *mcr-1* sequences deposited in GenBank lack any copy of IS*Apl1*. However, there was considerable redundancy in these sequences, with just 8 unique insertion sites identified from the 181 different submissions (Table S1). Every sequence is plasmid borne, with IncI2 and IncX4 plasmids being the most common groups (60% and 36%, respectively). Similarly to the single-ended variants, the sequences encompassing *mcr-1* and *pap2* are >99% identical except for a concentration of base changes at both the 5′ and 3′ ends of the region (Fig. 4). We conducted the same analysis of these sequences as for the single-ended variants described above. In all examples, as seen with the loss of the upstream IS*Apl1*, the loss of the downstream IS*Apl1* copy could be explained in a manner identical to that employed for the single-ended variants.

Figure 6 depicts multiple examples of the IRL and IRR and surrounding sequences of the upstream IS*Apl1* from a variety of insertion sites and plasmids. The composite transposon MTY17668-MCR1.5 is an example of the ancestral sequence that gave rise to the double-deletion variants (see below). In addition, we inferred the sequence of hypothetical parental single-ended variants for pH226B and pMCR16_PO53 (marked with an asterisk), as no corresponding single-ended variants of these double-deletion structures have been deposited to date. The 2-bp TSDs flanking the hypothetical upstream IRL (IE) that would have been generated upon insertion of the original composite transposon (prior to the loss of the upstream copy) are indicated (black, solid rectangle above the dinucleotide). The conserved, ancestral AT dinucleotide abutting the IRR (IE) of the upstream IS*Apl1* is also indicated (black triangle). The aligned sequences presented at the bottom of Fig. 6 illustrate the actual double-deletion variants formed through the loss of the upstream IS*Apl1* in the real and “hypothetical” single-ended variants described above. The deletion joint is encased in a black rectangle and highlighted in green. Nucleotides highlighted in red represent the original sequence upstream of the excision point retained in the double deletion variant, while those highlighted in blue represent the retained downstream sequence. The corresponding empty site for each sequence is highlighted in gray, with the 2-bp TSD that would have been generated upon insertion of the hypothetical ancestral Tn*6330* highlighted in black (solid rectangle above the dinucleotides). As noted for the single-ended variants, the nucleotides that form the basis for the variation at the 5′ end of the double-deletion structures (highlighted in bold) can be consistently explained by the presence of small variations in the region excised during loss of the upstream IS*Apl1* and the point at which the two ends are joined after the excision. Therefore, loss of the upstream copy appears to occur in a way similar to that seen with the downstream IS*Apl1* copy.

### *mcr-1*-IS*Apl1*: single-ended Tn*6330* variants were created by the loss of an upstream IS*Apl1.*

In addition to the three general *mcr-1* structures identified previously (Fig. 1A, B, and C) (12), we also identified a rare fourth structure that includes only a downstream IS*Apl1* copy (Fig. 1D). Only three such sequences were available. They represent 2 unique insertion sites (Table S1) in IncI2 and IncX4 plasmids. In two of the structures (KY363997 and KY363999), IS*1A* has a 59-bp sequence inserted upstream of the *mcr-1* gene, creating a characteristic IS*1A* 9-bp TSD (AAAAAATTG). Analysis performed using the procedures outlined above with corresponding empty sites (and intact Tn*6330* insertions) demonstrated that loss of the upstream IS*Apl1* copy can also explain these structures (data not shown).

### The demise of Tn*6330*.

**FIG 7:**
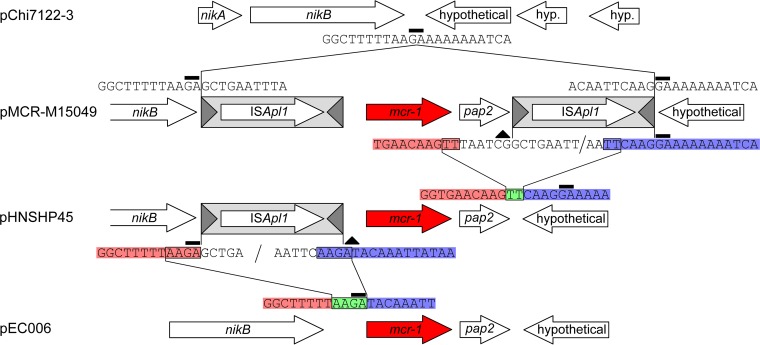
Alignment showing the formation and successive decay of Tn*6330* in a set of four highly related IncI2 plasmids. The labeling scheme is identical to that described for Fig. 3.

We identified a set of related incompatibility group IncI2 plasmids that clearly showed consecutive *in situ* stages in Tn*6330* acquisition and decay (Fig. 7). pChi7122-3 is an example of an IncI2 plasmid with an empty site. Insertion of Tn*6330* into this plasmid at a GA dinucleotide, next to *nikB* (relaxase gene), generated a close relative of pMCR-M15049 and a 2-bp GA TSD. Deletion of a sequence between a TT dinucleotide (encased in a black rectangle in the figure) within the downstream IS*Apl1* IRR and a second upstream TT (encased in a black rectangle) between the end of the *pap2* gene and the IRL of the IS copy yielded a close relative of pHNSHP45 that formed when the ends (highlighted in red and blue) joined at the TT dinucleotide (highlighted in green and encased in a black rectangle) after excision of the downstream IS*Apl1*. A subsequent excision of the upstream IS*Apl1* occurred between an AAGA tetranucleotide (encased in a black rectangle) abutting the upstream IS*Apl1* IRL and a second AAGA tetranucleotide (encased in a black rectangle) spanning the IRR. This generated a close relative of plasmid pSLy1 formed when the ends (highlighted in red and blue) joined at the AAGA tetranucleotide (highlighted in green and encased in a black rectangle), and that was thus devoid of both flanking IS*Apl1* copies. Notably, the plasmid backbones of pMCR-M15049, pHNSHP45, and pEC006 differ by only 10 to 11 single nucleotide polymorphisms (SNPs), strongly suggesting that these plasmids indeed represent successive periods of decay of Tn*6330* as a consequence of loss of IS*Apl1*.

Together, these results provide a convincing scenario explaining the sequestration of the *mcr-1* gene into an IS*Apl1*-based composite transposon, Tn*6330*, from a genome similar to that of *Moraxella* sp. MSG13-C03 and the transmission of this transposon to various plasmid replicons and its decay into a series of nontransposable derivatives.

## DISCUSSION

A comprehensive analysis of 273 intact *mcr-1* sequences representing 38 unique insertion sites (see Table S1 in the supplemental material) allowed us to formulate a model for the formation and subsequent decay of Tn*6330*, the primary vehicle for the mobilization of colistin resistance gene *mcr-1* (13, 14). A previous analysis of an initial set of *mcr-1* sequences revealed that *mcr-1* was part of an ~2,609-bp region flanked by one copy, two copies, or no copies of IS*Apl1* (12). In this report, we demonstrate that all *mcr-1* structures can be explained by loss of one or both copies of IS*Apl1* from an ancestral Tn*6330* and provide a scenario that maps this course of decay.

Recently, a 2,606-bp chromosomal region containing *mcr-1.10* from *Moraxella* sp. MSG13-C03 was identified which showed ~97% nucleotide identity with the 2,609-bp *mcr-1* region from Tn*6330* (Fig. 2) (11). The region contains 3 indels compared to the *mcr-1* structure, but the missing bases are all in noncoding regions (Fig. 2, colons). Importantly, this 2,606-bp region is flanked by the ancestral dinucleotide IS*Apl1* target sites (upstream, AT; downstream, CG) (Fig. 2, bold text) previously identified at the inside ends (IE) of the flanking IS*Apl1* copies in Tn*6330*. Although we have yet to identify presumed intermediates in Tn*6330* formation (i.e., the chromosomal target *mcr-1* region with a single upstream or downstream IS*Apl1* insertion), the presence of the conserved dinucleotide embedded in consensus IS*Apl1* target sequences together with the extremely high level of identity between the *Moraxella* sp. MSG13-C03 chromosome sequence and that of Tn*6330* leave little doubt as to the ancestry of Tn*6330*.

In the current library, there are 30 examples of complete Tn*6330* copies, 23 of which exhibited 2-bp TSDs; 59 examples of structures with an upstream IS*Apl1*; only 3 examples with a downstream IS*Apl1*; and 180 examples with no associated IS (Table S1). Analysis of the *mcr-1* sequence environment presented here strongly supports the idea that, once acquired, Tn*6330* has a strong tendency to decay by undergoing deletion which removes part or all of both downstream and upstream IS copies, resulting in an immobile *mcr-1* region trapped in its vector plasmid (Fig. 5 and 6).

Ten structures with an upstream IS copy also included a small segment of the downstream IS copy with the entire right downstream IS*Apl1* terminal IR (IRR). These were flanked by a 2-bp duplication typical of the target site duplication (TSD) generated by IS*30* family members. This suggested that these might be so-called “single-ended” transposons that have been found with different resistance genes and other IS types (4, 5, 23). These sequences represent 5 unique insertion sites (Table S1) and are potentially competent in transposition. In other examples, the downstream IS segment was absent and a sequence showing only limited similarity to IRR, but possessing a potential 2-bp TSD, was present. This raised the formal possibility that these structures could continue to transpose (12, 15, 16, 24). A third type of structure contained the *mcr-1* region but no accompanying IS*Apl1* sequences.

That the structures with only an upstream IS*Apl1* might transpose stemmed from the observations that some are flanked by typical IS*30* family consensus target sequences (12), that they possess an apparent TSD (12), and that, in some, a short sequence abutting the potential TSD resembled the terminal IRR of IS*Apl1*. Analysis of the upstream and downstream sequences in the extended library suggested that all these examples were generated by deletion between a downstream IS*Apl1* IRR and a deletion joint located in the 3′ end of the *pap2* gene (Fig. 4B). A comparison of these derivatives with Tn*6330* and empty sites revealed a “ragged” junction between the 3′ end of the *mcr-1* region and the area immediately adjacent to the downstream IS*Apl1* (Fig. 5). The junctions varied from isolate to isolate, implying that the downstream IS*Apl1* has repeatedly undergone a form of imprecise excision to generate this degree of sequence diversity. Similar conclusions could be drawn for the three examples in which the downstream IS was retained but the upstream was deleted (Table S1). Such behavior had previously been observed for IS*30* itself under controlled laboratory conditions (17).

**FIG 8:**
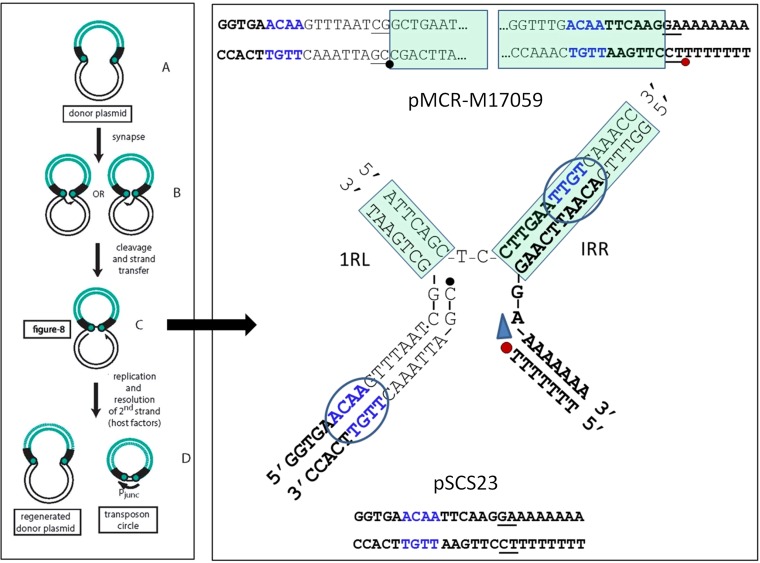
A mechanism for IS*Apl1* deletion. The left hand panel shows part of the IS*Apl1* transposition cycle. The IS is shown in green and the donor plasmid backbone in black. The terminal IRs are indicated by black boxes and the tips of the IS as green circles. (A to C) The IS ends in the donor plasmid (A) undergo synapsis (B), and one or other end undergoes cleavage to generate a 3′OH which then attacks the opposite end at a position several nucleotides into the donor backbone to generate a molecule in a figure eight formation in which the two IRs are joined by a single-strand bridge (C). (D) A 3′OH generated on the donor plasmid is then used to replicate the IS, generating a double-strand circular transposition intermediate. A strong promoter is generated by the juxtaposition of the two IS ends which drives high levels of transposase expression, facilitating insertion into a suitable target DNA (not shown). The right panel (top) shows the double-strand sequence of the IS ends in IncI2 plasmid pMCR-M17059 as an example. The middle panel presents the structure of the single-strand bridged molecule (as described for panel C) that is shown in the left panel. IS ends are boxed in green. The 3′OH generated in the donor plasmid DNA is indicated by a red dot and the corresponding 5′ phosphate at the other IS end by a black dot. The blue arrow indicates the direction of transposition-associated replication. The deletion joint is shown in blue. The sequence remaining after deletion (bottom) representing plasmid pSCS23 is composed of the bold black characters together with one of the blue tetranucleotide sequences.

In all cases, deletion involves flanking nucleotides ranging in size from 1 to 4 bp, and a single copy of these remains at the deletion junction (Fig. 5, 6, and 7; highlighted in green). One way of understanding how these events might occur is suggested by the “copy-out–paste-in” transposition mechanism first demonstrated for IS*3* family member IS*911* (25) and also shown to apply to members of the IS*30* family (18) (Fig. 8). A first chemical step in this pathway is cleavage of one end of the IS to generate a 3′OH. In a second step, the free 3′OH attacks the opposite IS end at a small distance (generally the length of the TSD), creating a single-strand bridge (Fig. 8, left) and generating a 3′OH on the donor flank. Note that we have indicated a 2-bp sequence between the abutted IRs (26) (Fig. 8, right) although 1-bp and 3-bp spacers have also been reported (A. Arini, M. P. Keller, W. Arber, unpublished data; cited in reference 17). The 3′OH is then used as a primer in replication (27) to generate a circular IS copy that subsequently integrates into a suitable target. The bridged intermediate shown in Fig. 8 uses the sequence of plasmid pMCR-M17059 (IncI2) as an example and shows how pSCS23 (Fig. 5) might have been generated by template switching during the copy-out transposition replication step. The observation that the vast majority of IS*Apl1* deletions have a length similar to that of the IS would support a model involving anchoring the two IS ends in close proximity (Fig. S2) followed by abortive transposition in which replication would fail to traverse the IS and generate the circular transposition intermediate.

Szabó et al. observed with IS*30* that this type of deletion occurred as a minor component of all deletions (17). However, they observed similar products, albeit at a 10^3^-fold-lower frequency, when an IS*30* derivative with an ablated transposase gene was used. However, in these cases, the vast majority of events proved to be more complex and included large deletions or unidentified plasmid rearrangements, suggesting that the spectrum of events was different in the absence of a functional transposase.

It is interesting that the single-ended Tn*6330* variants that retain an intact IRR segment of the ancestral downstream IS*Apl1* were generated by deletions whose lengths were significantly different from those of the typical 1,070-bp deletions noted for the other variants: 1,030 bp and 979 bp in the 42-bp and 90-bp variants, respectively (Fig. 3). Analysis of these ends revealed that the lengths of the flanking nucleotides where the two strands are joined after IS*Apl1* excision increased to 7 bp and 10 bp in the 42-bp and 90-bp variants, respectively, offering a possible reason for the existence of these structures (Fig. 3).

We are aware that the flanking sequences giving rise to deletion joints in certain deletion events are not robust and can be as short as a single base pair. However, on the basis of the single-strand bridged structure (Fig. 8), it seems possible that these sequences are not essential but simply assist in resolving a template switching event provoked by an error at the replicative (copy-out) transposition step. This would therefore reflect nonproductive transposition. In this light, if IS*Apl1*, like IS*30*, can generate spacers of 1 and 3 nucleotides (nt) as well as the typical 2-nt bridge (Arini et al., unpublished; cited in reference 17), it is possible that intermediates with atypical spacers which would change the architecture of the bridged intermediate are less efficient in the “copy-out” process and that it is these that increase the propensity for template switching. With the present facility in DNA sequencing, it would be useful to revisit these issues to determine the host factors involved in this behavior *in vivo*.

It has been pointed out by Szabó et al. that such deletions “can result in fixation of marker genes” by preventing further transposition (17). IS*Apl1* appears to be extremely active and was observed to undergo robust transposition in serial *mcr-1*-positive *Escherichia coli* isolates from a patient over a period of a month (28). Deletion of the flanking IS*Apl1* copies would therefore prevent further plasmid rearrangements.

**FIG 9:**
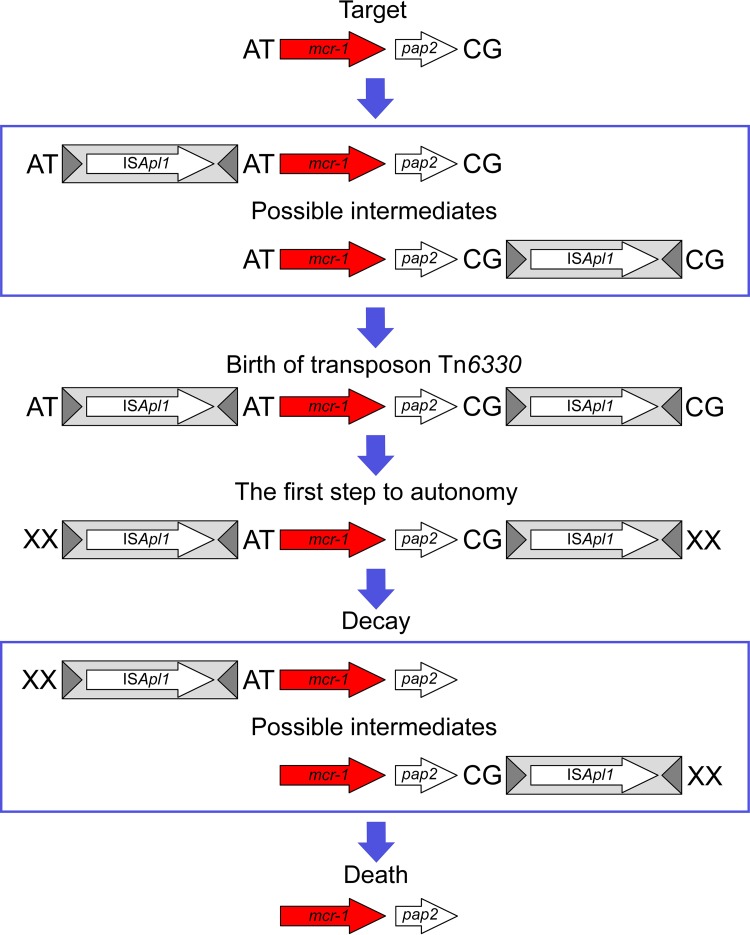
The birth and demise of Tn*6330*. A schematic representation of the birth of Tn*6330* following insertion of two copies of IS*Apl1* into the chromosome of a species closely resembling *Moraxella* sp. MSG13-C03 followed by successive decay of the transposon due to the loss of one IS*Apl1* copy and then both IS*Apl1* copies is shown. The conserved AT and CG dinucleotides that were formed during the first sequestration of the *mcr-1* region, and that are now found on the IE of all instances of Tn*6330*, are highlighted in bold.

In summary, the results presented here provide a strong framework for understanding how a chromosomal copy of the *mcr-1* gene may have been sequestered by IS*Apl1* to form the composite transposon Tn*6330* and how this transposon had subsequently decayed by loss of the upstream and downstream IS copies, reducing the probability of plasmid destruction by IS*Apl1*-mediated rearrangement and also resulting in a stabilized (nontransposable) copy of the resistance gene (Fig. 9).

## MATERIALS AND METHODS

All *mcr-1*-containing sequences deposited in GenBank through September 2017 were downloaded from the nucleotide and Whole Genome Shotgun (WGS) databases. Only entries with >1,000 bp of flanking sequence on both ends of the *mcr-1* gene (*n* = 273) were subjected to further analysis. Comparative genomic analysis was performed using the Geneious 8 software package (Biomatters Ltd., Auckland, New Zealand). Sequence features were annotated using the Geneious 8 annotation transfer tool, and sequences were aligned using a combination of BLAST (29), MUSCLE (30), progressive MAUVE (31), and LASTZ alignment tools. Average nucleotide identity data were calculated using BLAST (ANIb) and JSpecies (32), core genome alignments were performed using PanSeq (33), and phylogenetic trees were constructed using RAxML (34).
