# Supplementary figures and images for: The Birth and Demise of the ISApl1-mcr-1-ISApl1 Composite Transposon: the Vehicle for Transferable Colistin Resistance
Source: mBio. 2018 Feb 13;9(1):e02381-17. doi: 10.1128/mBio.02381-17 (PMC5821093; doi:10.1128/mBio.02381-17)

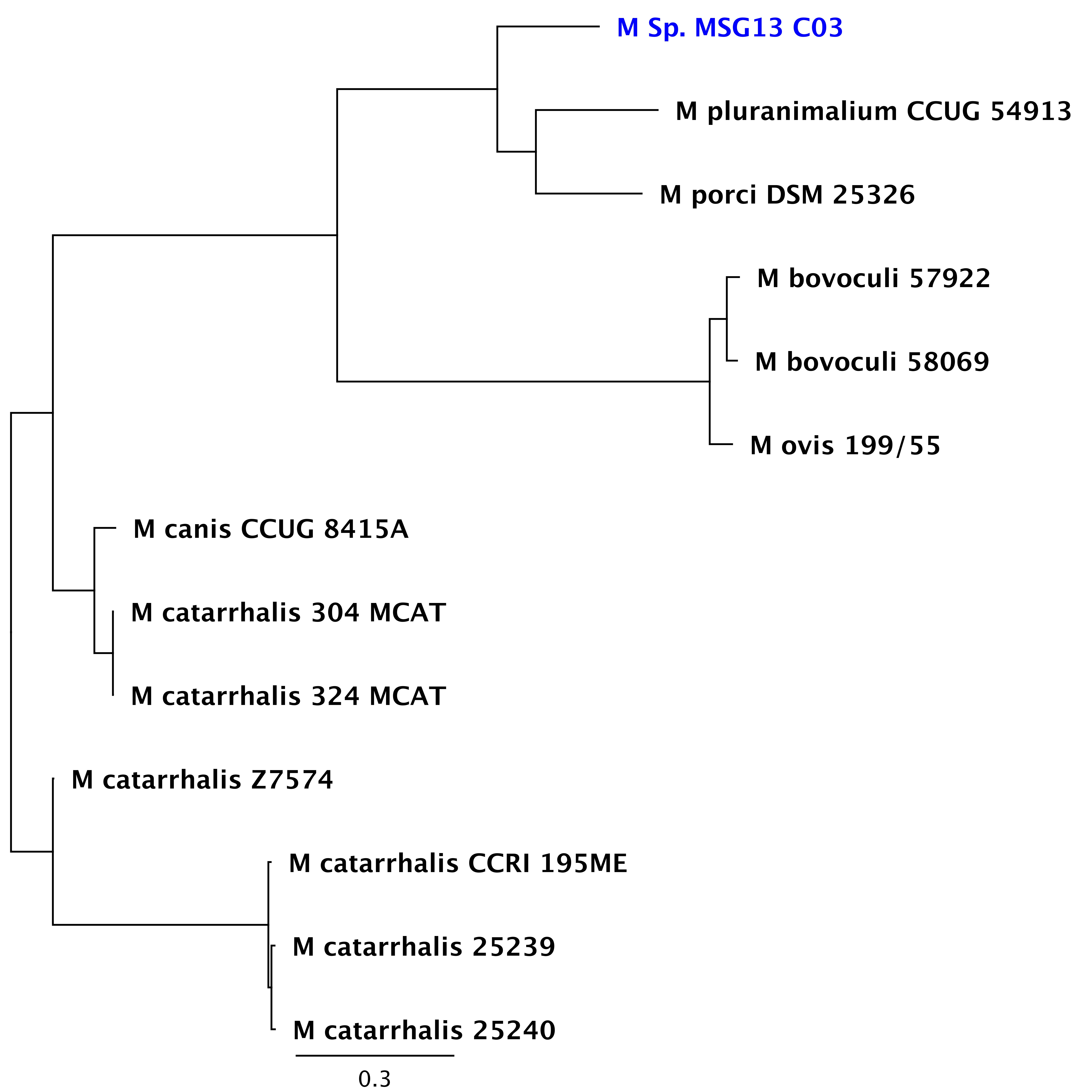

Supplement: FIG S1 [file mbo001183706sf1.tif]

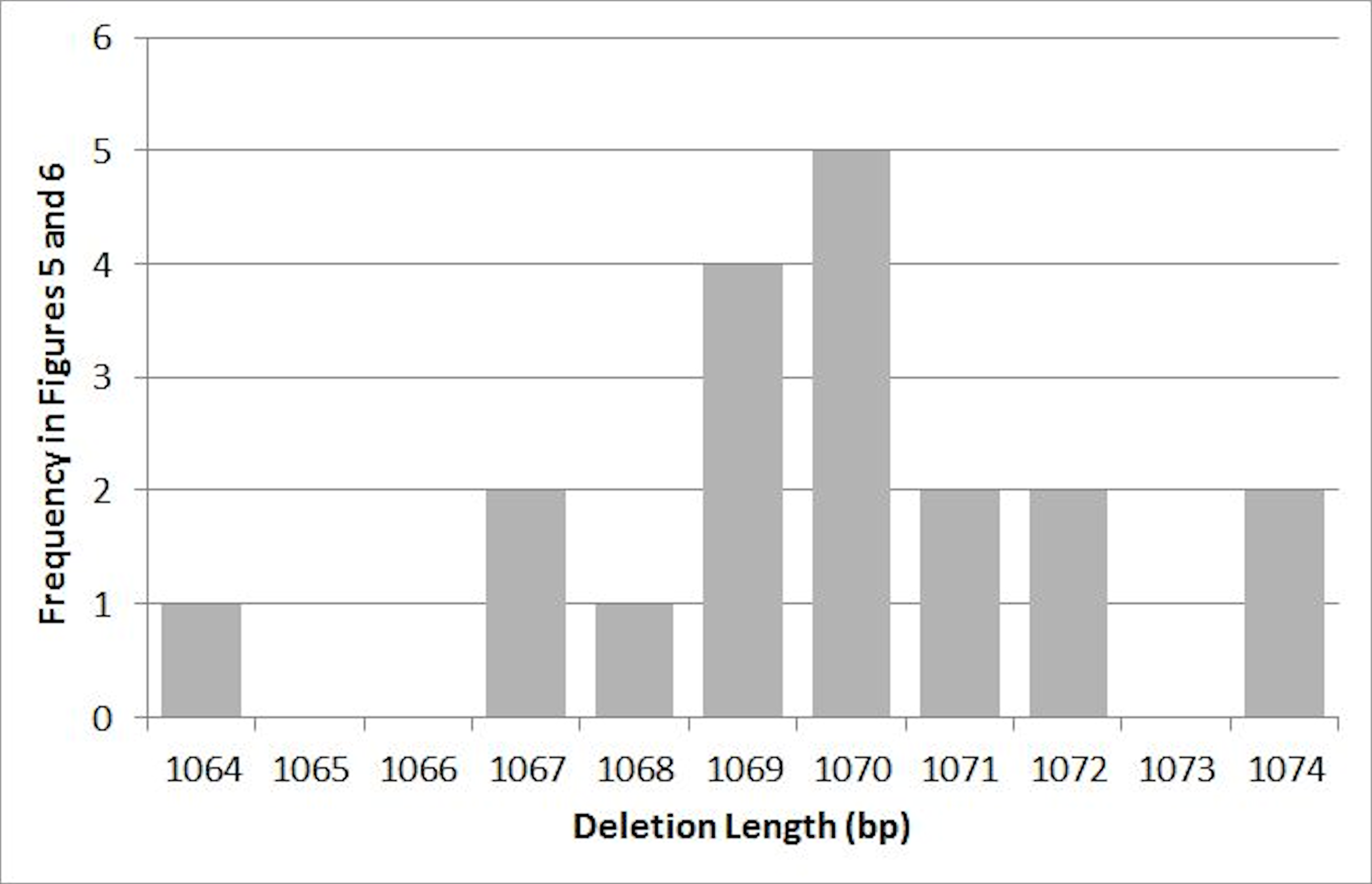

Supplement: FIG S2 [file mbo001183706sf2.tif]
